# ALDH1 is an immunohistochemical diagnostic marker for solitary fibrous tumours and haemangiopericytomas of the meninges emerging from gene profiling study

**DOI:** 10.1186/2051-5960-1-10

**Published:** 2013-05-09

**Authors:** Corinne Bouvier, François Bertucci, Philippe Métellus, Pascal Finetti, André Maues de Paula, Fabien Forest, Karima Mokhtari, Catherine Miquel, Daniel Birnbaum, Alexandre Vasiljevic, Anne Jouvet, Jean-Michel Coindre, Anderson Loundou, Dominique Figarella-Branger

**Affiliations:** 1 Department of Neuropathology and APHM Tumor Bank, LaTimone Hospital, Marseille, France; 2 UMR911, Equipe 4, Faculté de Médecine de Marseille, 27 Boulevard Jean Moulin, Marseille Cedex 513385, France; 3 Department of Medical Oncology, Institut Paoli-Calmettes, UMR891 Inserm, Aix Marseille University, Marseille, France; 4 Department of Molecular Oncology, Institut Paoli-Calmettes, UMR891 Inserm, Aix Marseille University, Marseille, France; 5 Department of Neurosurgery, La Timone Hospital, UMR911, Aix Marseille University, Marseille, France; 6 Department of Neuropathology, La Pitié Salpétrière Hospital, Paris, France; 7 Department of Neuropathology, Sainte Anne Hospital, Paris, France; 8 Department of Pathology, Wertheimer Hospital, Lyon, France; 9 Department of Pathology, Institut Bergonié, Bordeaux, France; 10 Department of Public Health, Faculty of Medicine and Marseilles University Hospital System (AP-HM), Marseille, France

**Keywords:** Solitary fibrous tumours, Haemangiopericytomas, Meningiomas, Synovial sarcomas, ALDH1, Gene expression profile, Immunohistochemistry

## Abstract

**Background:**

Solitary Fibrous Tumours (SFT) and haemangiopericytomas (HPC) are rare meningeal tumours that have to be distinguished from meningiomas and more rarely from synovial sarcomas. We recently found that *ALDH1A1* was overexpressed in SFT and HPC as compared to soft tissue sarcomas. Using whole-genome DNA microarrays, we defined the gene expression profiles of 16 SFT/HPC (9 HPC and 7 SFT). Expression profiles were compared to publicly available expression profiles of additional SFT or HPC, meningiomas and synovial sarcomas. We also performed an immunohistochemical (IHC) study with anti-ALDH1 and anti-CD34 antibodies on Tissue Micro-Arrays including 38 SFT (25 meningeal and 13 extrameningeal), 55 meningeal haemangiopericytomas (24 grade II, 31 grade III), 163 meningiomas (86 grade I, 62 grade II, 15 grade III) and 98 genetically confirmed synovial sarcomas.

**Results:**

*ALDH1A1* gene was overexpressed in SFT/HPC, as compared to meningiomas and synovial sarcomas. These findings were confirmed at the protein level. 84% of the SFT and 85.4% of the HPC were positive with anti-ALDH1 antibody, while only 7.1% of synovial sarcomas and 1.2% of meningiomas showed consistent expression. Positivity was usually more diffuse in SFT/HPC compared to other tumours with more than 50% of tumour cells immunostained in 32% of SFT and 50.8% of HPC. ALDH1 was a sensitive and specific marker for the diagnosis of SFT (SE = 84%, SP = 98.8%) and HPC (SE = 84.5%, SP = 98.7%) of the meninges. In association with CD34, ALDH1 expression had a specificity and positive predictive value of 100%.

**Conclusion:**

We show that ALDH1, a stem cell marker, is an accurate diagnostic marker for SFT and HPC, which improves the diagnostic value of CD34. ALDH1 could also be a new therapeutic target for these tumours which are not sensitive to conventional chemotherapy.

## Background

Solitary Fibrous Tumours (SFT) are ubiquitous mesenchymal tumours. Carneiro et al. reported the first cases occurring in the CNS in 1996 [1]. They usually arise in the meninges, though a few cases have been described without dural attachment. The histological features encompass a patternless architecture with alternating hypocellular and hypercellular areas. The hypocellular areas typically contain thick bands of collagen, while the hypercellular areas show staghorn vessels leading to a haemangiopericytoma (HPC) pattern. In soft tissues, HPC is thought to be the cellular variant of SFT [2,3]. However, SFT and HPC are still listed as separate entities in the WHO classification of CNS tumours [4,5] though we have reported their overlapping histological and immunohistological features, suggesting a common spectrum [6]. In the CNS, SFT have to be especially distinguished from meningiomas while HPC have to be distinguished from meningeal sarcomas. SFT lack the whorls, psammomas, nuclear-cytoplasmic inclusions and fibrous vessels present in meningiomas. The nuclei are more hyperchromatic. Slit-like vascular spaces are more common in SFT, but sometimes cellular elongation and collagen production may simulate a fibrous meningioma. More rarely, meningeal HPC can be confused with a sarcoma harboring a haemangiopericytoma pattern such as synovial sarcoma or mesenchymatous chondrosarcoma. Synovial sarcomas are characterized by a tumor-specific translocation t(X;18)(p11.2;q11.2) leading to the SS18-SSX fusion gene [7-9]. The presence of cartilaginous islands is the obvious distinctive feature of mesenchymal chondrosarcoma. By immunohistochemistry (IHC), SFT lack EMA expression, whereas they are usually diffusely positive for CD34 as are meningeal HPC. Synovial sarcomas and mesenchymal chondrosarcomas do not usually express CD34. On the other hand, meningiomas can only react to a limited extent with CD34 and widely express EMA. Thus, the CD34 positivity of SFT is non-specific and non-constant, calling for the identification of more accurate diagnostic molecular markers.

Using whole-genome oligonucleotide microarray, we recently identified a list of 3,401 genes differentially expressed between SFT/HPC (n = 29) and Soft Tissue Sarcomas (STS) (n = 179), including synovial sarcomas (Bertucci et al., manuscript in preparation). Among them, the *ALDH1A1* gene was among the top two genes overexpressed in SFT/HPC with a fold change (FC) between mean expression levels in SFT/HPC *versus* STS equal to 34. ALDH1 is a cytosolic detoxifying enzyme responsible for the oxidation of intracellular aldehydes. It is also highly expressed in hematopoietic progenitors where it is linked to retinoid metabolism with a role in early differentiation of stem cells. In search of a diagnostic value, we have performed an immunohistochemical study with antibody anti-ALDH1 in 243 meningeal tumours that encompassed: 25 CNS WHO SFT, 55 CNS WHO HPC and 163 meningiomas on Tissue MicroArrays. This was compared to ALDH1 expression of 98 synovial sarcomas and 13 extrameningeal SFT. We tested the hypothesis that ALDH1 expression could help to distinguish meningeal SFT or HPC from meningiomas or synovial sarcomas. We compared its expression at both the mRNA and protein levels using respectively gene expression data and IHC data on Tissue MicroArrays (TMA). We also compared ALDH1 immunohistochemical expression to CD34, the most consistently expressed antigen in SFT and HPC.

## Results

### *ALDH1A1* mRNA is overexpressed in SFT/HPC as compared to synovial sarcomas and meningiomas

**Figure 1:**
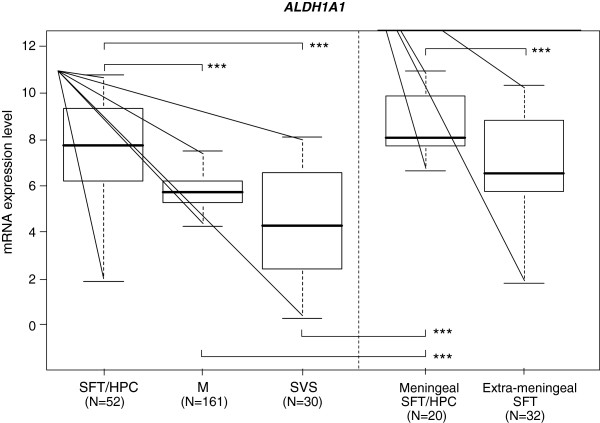
**mRNA expression of *ALDH1A1 *in tumour samples.***ALDH1A1 *expression across tumor samples profiled using DNA microarrays. Log_2_-transformed expression levels in tumors are reported as a box plot according to histological type: SFT, solitary fibrous tumour; HPC, Haemangiopericytoma; M, meningioma; SVS, synovial sarcoma; meningeal SFT and extra-meningeal SFT. Median and ranges are indicated. *** means p < 0.001 (t-test).

Normalised RNA expression level of *ALDH1A1* was available for 52 SFT/HPC, 161 meningiomas and 30 synovial sarcomas. As shown in Figure 1. *ALDH1A1* expression was significantly higher in SFT/HPC as compared with synovial sarcomas (p = 9.4E-08, t-test) with a mean fold change (FC) equal to 9.5. Similarly, *ALDH1A1* expression was significantly higher in SFT/HPC as compared with meningiomas (p = 1.8E-06, t-test) with a mean FC equal to 3.

Interestingly, *ALDH1A1* mRNA expression was higher in the 20 meningeal tumours than in the 32 extra-meningeal tumours (p = 7.5E-4; t-test). We thus repeated the comparative analysis using only meningeal tumours. Differences between meningeal SFT/HPC and other histological types were even more significant than before (Figure 1); the FC between SFT/HPC and synovial sarcoma increased to 20.2 (p = 1.2E-10, t-test) and between SFT/HPC and meningiomas increased to 6.4 (p = 6.7E-9, t-test).

Thus, at the mRNA level, SFT/HPC, and especially the meningeal locations, showed higher *ALDH1A1* expression than synovial sarcomas and meningiomas.

No significant difference was observed between SFT and HPC. By contrast, significant differences were observed between SFT/HPC and synovial sarcomas and meningiomas. We found similar results when analyses were applied to the 12 meningeal tumours only. Results are shown in Additional file 1: Table S2A and 2B.

### ALDH1 immunostaining

#### Solitary fibrous tumors and haemangiopericytomas (Table 1)

**Table 1:** ALDH1 IHC data for SFT (meningeal and extrameningeal) and meningeal HPC

		**ALDH1 negative**	**ALDH1 positive**	**ALDH1 5-10%**	**ALDH1 11-50**	**ALDH1 >50%**
Meningeal HPC	grade II n = 24	6	18	5	2	11
n = 55	grade III n = 31	2	29	5	7	17
Meningeal SFT		4	21	3	10	8
n = 25						
Extrameningeal SFT		2	11	3	4	4
n = 13						

**Figure 2:**
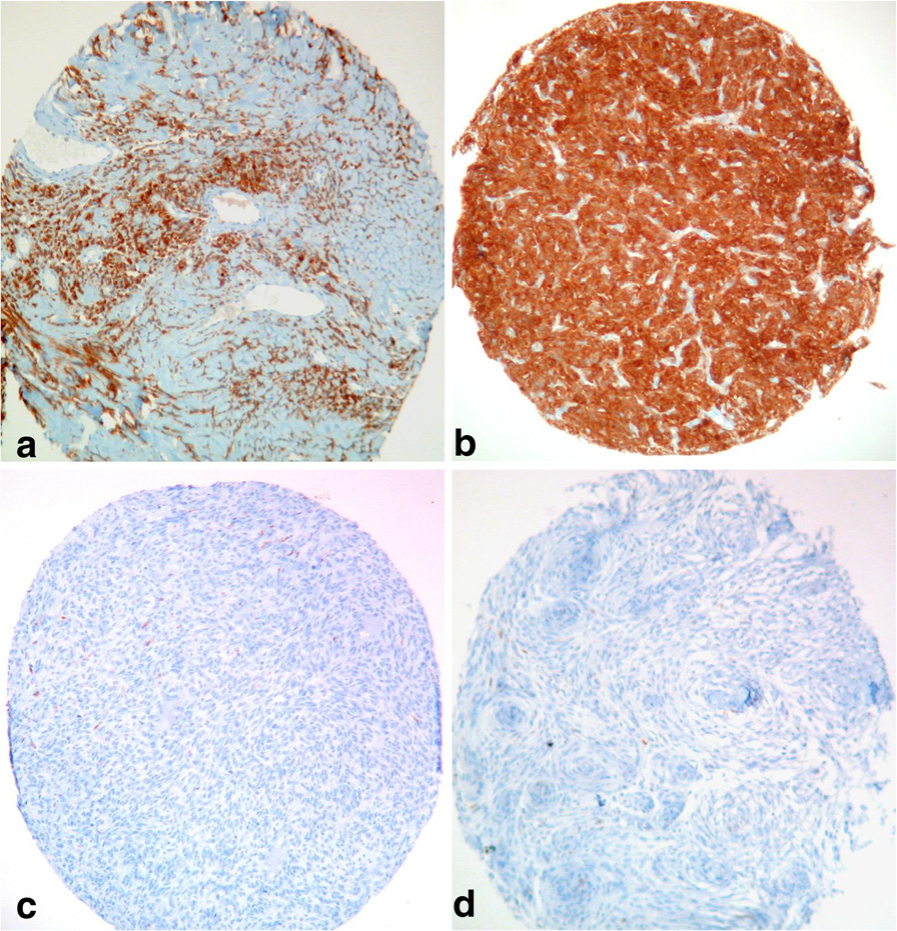
**ALDH1 immunohistochemistry on TMA (X10). **Diffuse expression of ALDH1 in a SFT (**a**) and meningeal HPC (**b**). No expression of ALDH1 in a monophasic fusiform cell synovial sarcoma (**c**) and a transitional Grade I meningioma (**d**).

84% of meningeal, 84.6% of extrameningeal SFT and 85.4% of HPC were positive with anti-ALDH1 antibody on TMA. Among HPC 75% of grade II and 93.5% of grade III were positive. Most SFT (72%) showed intermediate positivity (10 cases, 40%) or high positivity (8 cases, 32%) while 50.1% of HPC had high positivity (Figure 2a-b). ALDH1 immunostaining data were statistically not different between SFT and HPC though a tendency for having a more diffuse positivity was seen in grade III HPC (p = 0.118).

Protein expression by IHC was not statistically higher in meningeal tumours than extra-meningeal tumours, unlike what was observed at the mRNA level (Figure 1) (p = 1 for comparing positive and negative cases and p = 0.501 for semi-quantitative analysis).

#### Meningiomas and synovial sarcomas (Table 2)

**Table 2:** ALDH1 and CD34 data for meningeal SFT and HPC, meningiomas and synovialosarcomas

	**ALDH1+ CD34+**	**ALDH1+ CD34-**	**ALDH1- CD34+**	**ALDH1+ and or CD34+**	**ALDH1- CD34-**
SFT	20	1	3	24	1
n = 25					
Grade II HPC	14	4	5	23	1
n = 24					
Grade III HPC	20	9	2	31	0
n = 31					
meningiomas	0	2	8	10	153
n = 163					
Synovialosarcomas	0	7	0	7	91
n = 98					

Most meningiomas (98.8%) and synovial sarcomas (92.9%) lack ALDH1 expression by immunohistochemistry (Figure 2c-d). Among meningiomas, only two cases (1.2%) were positive for ALDH1. They were both grade II. One had low positivity, while the other one showed intermediate positivity. These cases were reviewed and showed typical histological features of fibroblastic meningiomas. They both expressed EMA. Seven synovial sarcomas (7.1%) expressed ALDH1 by IHC, with low positivity in 3 cases and high positivity in 4 cases.

### CD34 immunostaining

#### Solitary fibrous tumors and haemangiopericytomas (Table 2)

92% of meningeal SFT and 74.5 of meningeal HPC were positive for CD34.

#### Meningiomas and synovial sarcomas (Table 2)

Among meningiomas, 5 grade I samples and 3 grade II samples were CD34-positive (4.9%). Six were fibroblastic and 2 were psammomatous. Five had low positivity. Two had intermediate and one had high positivity. These three meningiomas were all fibroblastic and showed EMA positivity. CD34 immunoreactivity was never observed in synovial sarcomas.

### Diagnostic value of ALDH1 and CD34 immunostaining (Table 3)

**Table 3:** Impact value of ALDH1 and CD34 IHC for the diagnosis of meningeal SFT or HPC versus meningioma

	**SE**	**CI 95%**	**SP**	**IC 95%**	**PPV**	**IC 95%**	**NPV**	**IC 95%**
ALDH1								
TFS n = 25	84%	0.63-0.94	98.8%	0.95-0.99	91.3%	0.7-0.98	97.6%	0.93-0.99
HPC = 55	85.4%	0.73-0.93	98.7%	0.95-0.99	95.9%	0.84-0.99	95.2%	0.9-0.97
CD34								
TFS n = 25	92%	0.72-0.98	95%	0.9-0.97	74.2%	0.55-0.87	98.7%	0.94-0.99
HPC n = 55	74.5%	0.61-0.85	95%	0.9-0.97	83.7%	0.69-0.92	91.7%	0.86-0.95
ALDH1 and CD34+								
TFS n = 20	80%	0.58-0.92	100%	0.97-1	100%	0.79-1	97%	0.93-0.99
HPC n = 34	62%	0.48-0.74	100%	0.97-1	100%	0.87-1	88%	0.83-0.93

SE: sensitivity, PPV: positive predictive value.

SP: specificity, NPV: negative positive value.

We found that ALDH1 and CD34 were expressed in most meningeal SFT and HPC in contrast to meningiomas. As shown in Table 2, most SFT (20 of 25) and HPC (34 of 55) were positive for both markers, while no meningiomas or synovial sarcomas were. 4 additional SFT and 20 HPC were positive for ALDH1 or CD34. Only one case of SFT was negative for both markers. However, at recurrence the tumour showed ALDH1 and CD34 positivity. One additional grade II HPC was negative for the two markers. Both tumours had no SYT rearrangement on FISH analysis. Therefore 24/25 (96%) meningeal SFT and 54/55 (98%) meningeal HPC expressed ALDH1 and or CD34 whereas only 10 /163 (6%) meningiomas and 7/98 (7%) synovial sarcomas did.

No statistically significant difference was seen when comparing sensitivity (SE) and specificity (SP) of each immunohistochemical marker (ALDH1 or CD34), for the diagnosis of SFT versus meningioma (p = 0.617 for SE and p = 0.114 for SP) or HPC versus meningioma (p = 0.264 for SE and p = 0.114 for SP). However, ALDH1 had a tendency for being more sensitive than CD34 in the group of grade III HPC (p = 0.07).

We then tested the diagnostic value of combined ALDH1 and CD34 IHC staining. 94% of meningiomas and 93% of synovial sarcomas were negative for both markers while 80% of SFT and 62% of HPC were positive. We thus found that the double positivity for CD34 and ALDH1 was highly specific for the diagnosis of SFT, with a specificity and positive predictive value of 100%.

## Discussion

SFT are newly described tumours in the CNS. Diagnosis is based on histological features according to the WHO classification: such as “a pattern-less architecture characterized by a combination of alternating hypocellular and hypercellular areas separated from each other by thick bands of hyalinized somewhat keloidal collagen and branching haemangiopericytoma-like vessels” [5]. As the main location is the meninges, they have to be distinguished from other meningeal tumours, especially fibroblastic meningiomas. A high cellularity in some SFT and in HPC may also suggest a sarcoma, especially synovial sarcomas because of their “haemangiopericytoma” vascular pattern. We have performed the most extensive FISH study on TMA of meningeal SFT and HPC in search of SYT rearrangement. No rearrangement was found, suggesting that no misdiagnosed synovial sarcomas were included in the series. CD34 antigen is the most consistent immunohistochemical marker of SFT or HPC. However, it lacks specificity being expressed by endothelial cells, hematopoietic progenitor cells, and a lot of solid tumours such as gastrointestinal stromal tumours, vascular tumours, or dermatofibrosarcoma protuberans [10-17]. In addition, CD34 is variably expressed in SFT/HPC with some tumours showing focal or weak positivity or being negative [18-20]. Other immunohistochemical markers expressed by SFT such as bcl2, CD99, FXIII are less specific than CD34 [21].

Cytogenetic studies failed to show consistent recurrent abnormalities in SFT or HPC of any location. Very few transcriptomic studies of SFT/HPC are available in the literature [22,23]. We have performed an extensive meningeal SFT/HPC gene profiling study and showed that, at the mRNA level these tumours, and particularly meningeal, had higher *ALDH1A1* expression than synovial sarcomas and meningiomas. This was consistent with a previous study by Hajdu et al. who showed high mRNA expression of *ALDH1A1* in SFT including meningeal cases but no immunohistochemical study was performed. These results were further extended by IHC in a larger series of samples including 38 SFT (25 meningeal and 13 extra-meningeal), 55 HPC, 163 meningiomas and 98 synovial sarcomas. ALDH1 immunostaining was highly specific for meningeal SFT/HPC diagnosis as only 1.2% of meningiomas and 7.1% of synovial sarcomas were positive. Compared to CD34 staining, ALDH1 sensitivity, specificity, positive and negative predictive values were not statistically significantly different. However sensitivity of ALDH1 immunostaining was slightly better than CD34 (p = 0.07) especially for grade III HPC and could be especially useful in this category of tumours. More interestingly, the combined CD34 and ALDH1 expression by IHC was highly suggestive of the SFT or HPC diagnosis with a specificity and positive predictive value of 100%.

ALDH1A1 belongs to the ALDH enzymes, a family of evolutionarily conserved enzymes comprised of 19 isoforms that are localized in the cytoplasm, mitochondria or nucleus [24]. They are responsible for oxidizing aldehydes to carboxylic acids. ALDH1A1 is one of the 6 ALDH1 isoforms, the others being ALDH1A2, ALDH1A3, ALDH1B1, ALDH1L1, ALDH1L2 [24]. It was recently suggested that the different isoforms of ALDH could be tumour-specific with diagnostic implications. ALDH1A1 is predominantly expressed in the epithelium of testis, brain, eye, liver, kidney, neural and hematopoietic stem cells [25,26]. We have shown that *ALDH1A1* was overexpressed in SFT and HPC *versus* potential meningeal mimics. By immunohistochemistry ALDH1 was highly expressed in these tumours, however the antibody we used might not be entirely specific to the isoform ALDH1A1.

ALDH1 plays a major role in the biosynthesis of retinoic acid (RA) from retinol. It is this function that links ALDH1 to the “stemness” characteristics of the Cancer Stem Cells (CSC) since RA is a regulator of cellular proliferation, differentiation, apoptosis and survival. ALDH1 expression has been found in hematopoietic and epithelial cancers. Recently the presence of increased ALDH1 activity in a subpopulation of the human sarcoma cell line was also demonstrated [27,28]. These cells had stem cell properties since they were able to form spheres in anchorage-independent, serum-starved conditions, and showed high expression level of several stem cell genes such as Nanog, Oct3/4, Stat3 and Sox2. It is quite intriguing that the most consistent diagnostic markers for SFT and HPC, CD34 and ALDH1, are stem cell markers. These data could have histogenetic significance. The cell of origin of these tumors is unknown but is thought to be mesenchymal in nature [1]. High ALDH1 and CD34 expression also suggests an immature undifferentiated state.

In addition to its diagnostic interest, high ALDH1 expression in SFT and HPC also has therapeutic implications. It could be responsible in part for the failure of conventional chemotherapy, as has been suggested for other malignancies such as breast cancer [29-31], colon cancer [32] or medulloblastoma [33]. Indeed, ALDH1A1 is known to metabolize and detoxify chemotherapeutic agents such as cyclophosphamide [34] and ifosfamide [35]. On the other hand, ALDH1 emerges as a new therapeutic target as specific inhibitors of ALDH such as DEAB (4-diethylaminobenzaldehyde) exist [24,36].

Recently, two different teams identified recurrent *NAB2-STAT6* gene fusions on chromosome 12q13 in Solitary Fibrous Tumours of various locations [37,38]. Robinson et al. identified a *NAB2-STAT6* gene fusion in a hepatic metastasis of a malignant meningeal SFT by integrative sequencing. *NAB2-STAT6* gene fusion was also found in 27 additional SFT including 3 meningeal tumours [38]. Chmielecki et al. found 7 variants of this fusion transcript in 29/53 SFT (55%) [37]. *NAB2-STAT6* gene fusion is the first recurrent molecular feature reported in SFT. Further studies are required to confirm its diagnostic value especially it would be very interesting to compare the sensitivity and specificity of ALDH1 overexpression to *NAB2-STAT6* gene fusion. It would also be very interesting to search for *NAB2-STAT6* gene fusion in a large cohort of both meningeal SFT and HPC to definitively determine the relationship between these tumours.

## Conclusion

We have found that SFT and HPC displayed high *ALDH1A1* expression at the mRNA level compared to meningiomas and synovial sarcomas. By IHC, ALDH1 was an accurate marker for the diagnostic of SFT and HPC. This new marker had a high sensitivity and specificity that improves the diagnostic value of CD34. We recommend its use in routine practice in association with CD34.

## Methods

### Gene expression data sets of SFTs

To determine *ALDH1A1* mRNA expression in SFT/HPC, we first analyzed gene expression data recently generated by our laboratory (IPC, Marseille, France) from frozen tumor samples (APHM Biobank authorization number 2008/70, NeurobiotecBank, Sainte Anne Hospital CRB NSPN authorization number AC-2008-82, La Pitié Salpétrière Biobank) collected from 16 patients who underwent initial surgery and/or diagnostic biopsy. The origin of samples was meningeal in 12 cases (9 HPC and 3 SFT according to the CNS WHO classification) and extra-meningeal in 4 cases (3 conventional Soft tissue SFT and one cellular variant so called “HPC”). All profiled specimens contained more than 70% of cancer cells (as assessed before RNA extraction using frozen sections adjacent to the profiled samples). Each patient gave written informed consent for molecular analysis, and the study was approved by our institutional ethics committee. Expression profiles were established for these 16 samples with Affymetrix U133 Plus 2.0 human microarrays (Affymetrix^®^, Santa Clara, CA, USA) as previously described [39]. All data are MIAME compliant and the raw data will be publicly available after acceptance of the paper.

To increase the size of the SFT series, we collected two publicly available data sets: West’s set [23] collected from (http://www.ncbi.nlm.nih.gov/geo/query/acc.cgi?acc=GSE4305) and including 13 SFTs and 30 STS profiled using 42,000-element cDNA microarrays, and Hajdu’s set [22] collected from (http://cbio.mskcc.org/Public/SFT) and including 23 SFTs and 33 STS profiled using Affymetrix U133A microarrays. Whole-genome RNA expression profiles were thus available for 52 SFT/HPC samples, including 20 meningeal cases and 32 extra-meningeal cases. However, in the study of Hajdu et al., the meningeal cases encompassed 6 malignant SFT. Because of the rarity of this diagnosis in meningeal location some of these tumours might have the histological criteria of HPC according to the CNS WHO classification of brain tumours. So we used the terminology SFT/HPC for meningeal tumours.

### Gene expression data sets of synovial sarcomas and meningiomas

To analyze *ALDH1A1* mRNA expression in synovial sarcomas and meningiomas, we collected publicly available gene expression data sets. For meningiomas, we collected three data sets including a total of 161 samples: Claus’s set [40] collected from the National Center for Biotechnology Information (NCBI)/Genbank GEO database (series entry GSE9438) and including 31 samples, Lee’s set [41] collected from the GEO database (GSE16581) and including 68 samples, and Scheck’s set collected from the GEO database (GSE4780) and including 62 samples.

For synovial sarcomas, we collected four data sets including a total of 30 samples: Nakayama’s set [42] from the GEO database (GSE6481) and including 16 cases, Detwiller’s set [43] from the GEO database (GSE16581) and including 4 cases, West’s set [23] collected from (http://www.ncbi.nlm.nih.gov/geo/query/acc.cgi?acc=GSE4305) and including 6 cases, and Hajdu’s set [22] collected from (http://cbio.mskcc.org/Public/SFT) and including 4 cases.

### Analysis of gene expression data

All gene expression data sets are listed in Additional file 2: Table S1. RNA expression data were thus available for 52 SFT/HPC, 161 meningiomas and 30 synovial sarcomas, collected through 8 data sets. Before analysis, we first applied quantile normalization to the available processed West’s data set and MAS5-processed Detwiller and Nakayama’s Affymetrix data sets. For the other Affymetrix sets (raw data), we used Robust Multichip Average (RMA) [44] with the non-parametric quantile algorithm as normalization parameter. Quantile normalization or RMA was done in R using Bioconductor and associated packages.

Normalised expression data generated from different technological platforms and laboratories were then further processed to eliminate potential bias. Processing of data included as first step the selection of genes unique and common to all sets. We focused on *ALDH1A1* and a list of 575 human housekeeping genes [45]. Before the selection, we mapped hybridization probes across the different technological platforms (Affymetrix U133A and U133 Plus 2.0, and Stanford cDNA microarrays). Affymetrix gene chip annotations were updated using NetAffx Annotation files (http://www.affymetrix.com; release from 01/12/2008). Stanford gene annotations were retrieved and updated using EntrezGene (Homo sapiens gene information db, release from 09/12/2008, http://ftp.ncbi.nlm.nih.gov/gene/). All probes were then mapped on the basis of their EntrezGeneID. When multiple probes were mapped to the same GeneID, the one with the highest variance in a particular dataset was selected to represent the GeneID. For redundant Affymetrix probe sets, those with an extension « _at », next « s_at », and followed by all other extensions were preferentially kept. After mapping and selection, 451 out of the 575 housekeeping genes were retained. Among these 451 genes, we then selected the ones with the smallest variation in RNA expression across all data sets: 354 genes were retained, showing the smallest inter-set variance of intra-set variance, the cut-off of non-variance being defined by the distribution of variability measurements. The last step was the normalisation of all data sets based on 355 genes including the 354 housekeeping genes and *ALDH1A1* (Affymetrix probe set 212224_at, and IMAGE clone 855624 for West’s set) using Distance Weighted Discrimination (DWD) [46]. Principal Component Analysis (PCA) demonstrated that normalization was effective in removing technical, inter-laboratory variation in gene expression (data not shown), allowing us to compare *ALDH1A1* expression between all data sets. Comparison was done using continuous values.

Then, we compared (Mann–Whitney test) the *ALDH1A1* mRNA expression of HPC (N = 10) *vs* SFT (N = 6),of HPC (N = 10) *vs* synovial sarcomas (N = 30) and *vs* meningiomas (N = 161), and of SFT (N = 6) *vs* synovial sarcomas (N = 30) and *vs* meningiomas (N = 161).

### Immunohistochemistry

A total of 38 SFT (25 meningeal and 13 extrameningeal), 55 meningeal HPC, 163 meningiomas and 98 synovialosarcomas were selected for the IHC study. The eighty cases of SFT/HPC belonged to a previously published multicentric retrospective study [6]. The thirteen extra-meningeal SFT were located in soft tissues (9 cases) and the thorax (3 cases). The absence of t(X;18) in SFT samples was checked using a previously described FISH method [8] with the SS18 Break apart FISH probe Kit from Vysis:. 78 cases of 93 SFT were interpretable and did not show any SYT rearrangement. Meningiomas were retrieved from the pathological archives of the Timone Hospital. They included 86 grade I tumors, 62 grade II, and 15 grade III. Among the grade I there were 37 meningothelial, 27 fibroblastic, 14 transitional, 5 psamommatous, 2 secretory and one microcystic sub-types. A total of 98 genetically confirmed synovial sarcomas were provided from the pathological files of the The Institut Bergonié (Bordeaux).

Automate immunohistochemistry was performed on formol fixed paraffin embedded 4 μm sections from TMA paraffin blocks. All tumor specimens had been fixed in 4% formalin. TMAs were prepared as previously described [47]. Briefly, for each sample, three representative sample areas were carefully selected from a haematoxylin–eosin-stained section of a donor block, in order to punch core cylinders with a diameter of 0.6 mm (for SFT and meningiomas) and 1 mm for synovial sarcomas. Each tumour was punched from three representative areas and deposited into two separate recipient paraffin blocks using a specific arraying device (Alphelys). Automate IHC was performed using a Ventana automate (Benchmark XT, Ventana Medical Systems SA, Illkirch, France) and the two following antibodies: ALDH1 (clone 44/ALDH, immunogen Human ALDH1 aa. 7–128, BD Transduction Laboratories, dilution: 1/500), and CD34 (QBEND10, prediluted). One hour Heat antigen retrieval at 95° was used with a citrate buffer pH = 8.4 for ALDH1 and EDTA buffer pH = 8 for CD34. External positive control was a glioblastoma for ALDH1. For CD34, normal vessels serve as internal positive control. Negative controls corresponded to omission of primary antibody or irrelevant antibodies of the same isotype. IHC was recorded positive when cytoplasmic staining was observed in 5% or more of the cells for both markers. Then a semi-quantitative analysis was done for ALDH1-positive specimens. A mean of the percentage of each spot was recorded and classified as follows: low positivity = 5-10% of stained cells, intermediate positivity = 11-50%, high positivity > 50%.

### Statistical analyses

Comparison of *ALDH1A1* mRNA expression status according to histological type was done using the Student t-test applied to continuous values.

For immunohistochemistry, continuous variables are expressed as means ±SD or as median with range (min,max), and categorical variables are reported as count and percentages. Comparisons of means values between two groups were performed using student t-test or Mann–Whitney U. Comparisons of percentages were performed using Chi-Square test or (Fisher’s exact test, as appropriate). The discriminative ability of ALDH1 and CD34 immunostaining was quantified by the measures of diagnostic accuracy. All statistical tests were two-sided at the 5% level of significance. Statistical analysis was done using PASW Statistics version 17.02 (IBM SPSS Inc., Chicago, IL, USA) and R software. Sensitivity and specificity of ALDH1 and CD34 immunostainings were compared as previously reported [48].

## Authors’ contributions

CB and FB contributed equally to this work and should be considered as first coauthors. They designed the study, analyzed the results and wrote the paper. PM provided most surgical specimens. PF did the transcriptomic statistics. AMdP and FF did the SYT FISH. KM, CM, AV, AJ did the pathological diagnosis and provided paraffin embedded tumours. DB contributed to the transcriptome analysis. JMC provided synovial sarcoma tissue microarrays. AL did the statistics of immunohistochemical data. DFB coordonated the study and contributed to the writing of the paper. All authors read and approved the final manuscript.

## Competing interest

The authors declare that they have no conflict of interest.

## Supplementary Material

Additional file 1: Table S2 Comparison of ALDH1A1 mRNA expression (p-values; Mann-Whitney test) between SFT, HPC, meningiomas and synovial sarcomas. Click here for file

Additional file 2: Table S1 Description of gene expression data sets pooling SFT and HPC. Click here for file

## References

[B1] Carneiro SS Scheithauer BW Nascimento AG Hirose T Davis DH Solitary fibrous tumors of the meninges: a lesion distinct from fibrous meningioma. A clinicopathological and immunohistochemical study Am J Clin Pathol 1996 1 217 224 8712177 10.1093/ajcp/106.2.217

[B2] Gengler C Guillou L Solitary fibrous tumour and haemangiopericytoma: evolution of a concept Histopathology 2006 1 63 74 10.1111/j.1365-2559.2005.02290.x 16359538

[B3] Guillou L Fletcher JA Fletcher CDM Mandahl Fletcher CDM, Unni KK, Mertens F Extra pleural solitary fibrous tumour and haemangiopericytoma in WHO Classification of Tumours Pathology and Genetics of Tumours of Soft Tissue and Bone 2002 Lyon: IARC Press p86 p90

[B4] Giannini C Rushing EJ Hainfelier JA Louis DN, Ohgaki H, Wiestler OD, Cavenee WK Hemangiopericytoma in the WHO classification of Tumours of the Central Nervous System 2007 4 Lyon: IARC Press p178 p180

[B5] Paulus W Scheithauer BW Perry A Louis DN, Ohgaki H, Wiestler OD, Cavenee WK Mesenchymal non-meningothelial tumorsthe WHO classification of Tumours of the Central Nervous System 2007 4 Lyon: IARC Press p173 p177

[B6] Bouvier C Metellus P de Paula AM Vasiljevic A Jouvet A Guyotat J Mokhtari K Varlet P Dufour H Figarella-Branger D Solitary fibrous tumors and hemangiopericytomas of the meninges: overlapping pathological features and common prognostic factors suggest the same spectrum of tumors Brain Pathol 2012 1 511 521 10.1111/j.1750-3639.2011.00552.x 22082190 PMC8057625

[B7] Amary MF Berisha F Bernardi Fdel C Herbert A James M Reis-Filho JS Fisher C Nicholson NG Tirabosco R Diss TC Flanagan AM Detection of SS18-SSX fusion transcripts in formalin-fixed paraffin-embedded neoplasms: analysis of conventional RT-PCR, qRT-PCR and dual color FISH as diagnostic tools for synovial sarcoma Mod Pathol 2007 1 482 496 10.1038/modpathol.3800761 17334349

[B8] Lu YJ Birdsall S Summersgill B Smedley D Osin P Fisher C Shipley J Dual colour fluorescence in situ hybridization to paraffin-embedded samples to deduce the presence of the der(X)t(X;18)(p11.2;q11.2) and involvement of either the SSX1 or SSX2 gene: a diagnostic and prognostic aid for synovial sarcoma J Pathol 1999 1 490 496 10.1002/(SICI)1096-9896(199903)187:4<490::AID-PATH274>3.0.CO;2-X 10398111

[B9] Sandberg AA Bridge JA Updates on the cytogenetics and molecular genetics of bone and soft tissue tumors. Synovial sarcoma Cancer Genet Cytogenet 2002 1 1 23 10.1016/S0165-4608(01)00626-4 11890984

[B10] Aiba S Tabata N Ishii H Ootani H Tagami H Dermatofibrosarcoma protuberans is a unique fibrohistiocytic tumour expressing CD34 Br J Dermatol 1992 1 79 84 10.1111/j.1365-2133.1992.tb08036.x 1382538

[B11] Civin CI Strauss LC Brovall C Fackler MJ Schwartz JF Shaper JH Antigenic analysis of hematopoiesis. III. A hematopoietic progenitor cell surface antigen defined by a monoclonal antibody raised against KG-1a cells J Immunol 1984 1 157 165 6586833

[B12] Cohen PR Rapini RP Farhood AI Expression of the human hematopoietic progenitor cell antigen CD34 in vascular and spindle cell tumors J Cutan Pathol 1993 1 15 20 10.1111/j.1600-0560.1993.tb01243.x 7682226

[B13] Fina L Molgaard HV Robertson D Bradley NJ Monaghan P Delia D Sutherland DR Baker MA Greaves MF Expression of the CD34 gene in vascular endothelial cells Blood 1990 1 2417 2426 1693532

[B14] Lin G Finger E Gutierrez-Ramos JC Expression of CD34 in endothelial cells, hematopoietic progenitors and nervous cells in fetal and adult mouse tissues Eur J Immunol 1995 1 1508 1516 10.1002/eji.1830250606 7542195

[B15] Miettinen M Lindenmayer AE Chaubal A Endothelial cell markers CD31, CD34, and BNH9 antibody to H- and Y-antigens–evaluation of their specificity and sensitivity in the diagnosis of vascular tumors and comparison with von Willebrand factor Mod Pathol 1994 1 82 90 7512718

[B16] Nickoloff BJ The human progenitor cell antigen CD34 is localized on endothelial cells, dermal dendritic cells, and perifollicular cells in formalin-fixed normal skin, and on proliferating endothelial cells and stromal spindle-shaped cells in Kaposi’s sarcoma Arch Dermatol 1991 1 523 529 10.1001/archderm.1991.04510010091009 2006877

[B17] Traweek ST Kandalaft PL Mehta P Battifora H The human hematopoietic progenitor cell antigen (CD34) in vascular neoplasia Am J Clin Pathol 1991 1 25 31 1712541 10.1093/ajcp/96.1.25

[B18] Perry A Scheithauer BW Nascimento AG The immunophenotypic spectrum of meningeal hemangiopericytoma: a comparison with fibrous meningioma and solitary fibrous tumor of meninges Am J Surg Pathol 1997 1 1354 1360 10.1097/00000478-199711000-00010 9351573

[B19] Van de Rijn M Lombard CM Rouse RV Expression of CD34 by solitary fibrous tumors of the pleura, mediastinum, and lung Am J Surg Pathol 1994 1 814 820 10.1097/00000478-199408000-00008 7518652

[B20] Westra WH Gerald WL Rosai J Solitary fibrous tumor. Consistent CD34 immunoreactivity and occurrence in the orbit Am J Surg Pathol 1994 1 992 998 10.1097/00000478-199410000-00003 7522416

[B21] Hasegawa T Matsuno Y Shimoda T Hirohashi S Hirose T Sano T Frequent expression of bcl-2 protein in solitary fibrous tumors Jpn J Clin Oncol 1998 1 86 91 10.1093/jjco/28.2.86 9544821

[B22] Hajdu M Singer S Maki RG Schwartz GK Keohan ML Antonescu CR IGF2 over-expression in solitary fibrous tumours is independent of anatomical location and is related to loss of imprinting J Pathol 2010 1 300 307 10.1002/path.2715 20527023 PMC3264680

[B23] West RB Nuyten DS Subramanian S Nielsen TO Corless CL Rubin BP Montgomery K Zhu S Patel R Hernandez-Boussard T Goldblum JR Brown PO van de Vijver M van de Rijn M Determination of stromal signatures in breast carcinoma PLoS Biol 2005 1 e187 10.1371/journal.pbio.0030187 15869330 PMC1088973

[B24] Marcato P Dean CA Giacomantonio CA Lee PW Aldehyde dehydrogenase: its role as a cancer stem cell marker comes down to the specific isoform Cell Cycle 2011 1 1378 1384 10.4161/cc.10.9.15486 21552008

[B25] Armstrong L Stojkovic M Dimmick I Ahmad S Stojkovic P Hole N Lako M Phenotypic characterization of murine primitive hematopoietic progenitor cells isolated on basis of aldehydedehydrogenase activity Stem Cells 2004 1 7 1142 1151 10.1634/stemcells.2004-0170 15579635

[B26] Chute JP Muramoto GG Whitesides J Colvin M Safi R Chao NJ McDonnell DP Inhibition of aldehyde dehydrogenase and retinoid signaling induces the expansion of human hematopoietic stem cells Proc Natl Acad Sci USA 2006 1 31 11707 11712 10.1073/pnas.0603806103 16857736 PMC1544234

[B27] Honoki K Fujii H Kubo A Kido A Mori T Tanaka Y Tsujiuchi T Possible involvement of stem-like populations with elevated ALDH1 in sarcomas for chemotherapeutic drug resistance Oncol Rep 2010 1 501 505 20596639 10.3892/or_00000885

[B28] Wang L Park P Zhang H La Marca F Lin CY Prospective identification of tumorigenic osteosarcoma cancer stem cells in OS99-1 cells based on high aldehyde dehydrogenase activity Int J Cancer 2011 1 294 303 10.1002/ijc.25331 20309879

[B29] Ricardo S Vieira AF Gerhard R Leitao D Pinto R Cameselle-Teijeiro JF Milanezi F Schmitt F Paredes J Breast cancer stem cell markers CD44, CD24 and ALDH1: expression distribution within intrinsic molecular subtype J Clin Pathol 2011 1 937 946 10.1136/jcp.2011.090456 21680574

[B30] Sladek NE Kollander R Sreerama L Kiang DT Cellular levels of aldehyde dehydrogenases (ALDH1A1 and ALDH3A1) as predictors of therapeutic responses to cyclophosphamide-based chemotherapy of breast cancer: a retrospective study. Rational individualization of oxazaphosphorine-based cancer chemotherapeutic regimens Cancer Chemother Pharmacol 2002 1 309 321 10.1007/s00280-001-0412-4 11914911

[B31] Sreerama L Sládek NE Primary breast tumor levels of suspected molecular determinants of cellular sensitivity to cyclophosphamide, ifosfamide, and certain other anticancer agents as predictors of paired metastatic tumor levels of these determinants. Rational individualization of cancer chemotherapeutic regimens Cancer Chemother Pharmacol Mar 2001 1 3 255 262 10.1007/s002800000208 11320670

[B32] Rekha GK Sreerama L Sladek NE Intrinsic cellular resistance to oxazaphosphorines exhibited by a human colon carcinoma cell line expressing relatively large amounts of a class-3 aldehyde dehydrogenase Biochem Pharmacol 1994 1 1943 1952 10.1016/0006-2952(94)90593-2 7986206

[B33] Friedman HS Colvin OM Kaufmann SH Ludeman SM Bullock N Bigner DD Griffith OW Cyclophosphamide resistance in medulloblastoma Cancer Res 1992 1 5373 5378 1356617

[B34] Magni M Shammah S Schiró R Mellado W Dalla-Favera R Gianni AM Induction of cyclophosphamide-resistance by aldehyde-dehydrogenase gene transfer Blood 1996 1 3 1097 1103 8562935

[B35] Hingorani P Zhang W Piperdi S Pressman L Lin J Gorlick R Kolb EA Preclinical activity of palifosfamide lysine (ZIO-201) in pediatric sarcomas including oxazaphosphorine-resistant osteosarcoma Cancer Chemother Pharmacol 2009 1 733 740 10.1007/s00280-008-0922-4 19224214

[B36] Marchitti SA Brocker C Stagos D Vasiliou V Non-P450 aldehyde oxidizing enzymes: the aldehyde deshydrogenase superfamily Expert Opin Drug Metab Toxicol 2008 1 6 697 720 10.1517/17425255.4.6.697 18611112 PMC2658643

[B37] Chmielecki J Crago AM Rosenberg M O’Connor R Walker SR Ambrogio L Auclair D McKenna A Heinrich MC Frank DA Meyerson M Whole-exome sequencing identifies a recurrent *NAB2-STAT6* fusion in solitary fibrous tumors Nat Genet 2013 1 131 132 10.1038/ng.2522 23313954 PMC3984043

[B38] Robinson DR Wu MY Sundaram SK Cao X Lonigro R Sung YS Chen CL Zhang L Wang R Su F Iyer MK Roychowdhury S Siddiqui J Pienta KJ Kunju LP Talpaz M Mosquera JM Singer S Schuetze S Antonescu CR Chinnaiyan AM Identification of recurrent *NAB2-*STAT6 gene fusions in solitary fibrous tumor by integrative sequencing Nat Genet 2013 1 180 185 10.1038/ng.2509 23313952 PMC3654808

[B39] Bertucci F Finetti P Cervera N Charafe-Jauffret E Mamessier E Adélaïde J Debono S Houvenaeghel G Maraninchi D Viens P Charpin C Jacquemier J Birnbaum D Gene expression profiling shows medullary breast cancer is a subgroup of basal breast cancers Cancer Res 2006 1 9 4636 4644 10.1158/0008-5472.CAN-06-0031 16651414

[B40] Claus EB Park PJ Carroll R Chan J Black PM Specific genes expressed in association with progesterone receptors in meningioma Cancer Res 2008 1 1 314 322 10.1158/0008-5472.CAN-07-1796 18172325 PMC3256746

[B41] Lee Y Liu J Patel S Cloughesy T Lai A Farooqi H Seligson D Dong J Liau L Becker D Mischel P Shams S Nelson S Genomic landscape of meningiomas Brain Pathol 2010 1 4 751 762 20015288 10.1111/j.1750-3639.2009.00356.x PMC3167483

[B42] Nakayama R Nemoto T Takahashi H Ohta T Kawai A Seki K Yoshida T Toyama Y Ichikawa H Hasegawa T Gene expression analysis of soft tissue sarcomas: characterization and reclassification of malignant fibrous histiocytoma Mod Pathol 2007 1 7 749 759 10.1038/modpathol.3800794 17464315

[B43] Detwiller KY Fernando NT Segal NH Ryeom SW D’Amore PA Yoon SS Analysis of hypoxia-related gene expression in sarcomas and effect of hypoxia on RNA interference of vascular endothelial cell growth factor A Cancer Res 2005 1 13 5881 5889 10.1158/0008-5472.CAN-04-4078 15994966

[B44] Irizarry RA Hobbs B Collin F Beazer-Barclay YD Antonellis KJ Scherf U Exploration, normalization, and summaries of high density oligonucleotide array probe level data. Speed TP Biostatistics 2003 1 2 249 264 10.1093/biostatistics/4.2.249 12925520

[B45] Eisenberg E Levanon EY Human housekeeping genes are compact Trends Genet 2003 1 7 362 365 10.1016/S0168-9525(03)00140-9 12850439

[B46] Benito M Parker J Du Q Wu J Xiang D Perou CM Marron JS Adjustment of systematic microarray data biases Bioinformatics 2004 1 1 105 114 10.1093/bioinformatics/btg385 14693816

[B47] Salas S Jézéquel P Campion L Deville JL Chibon F Bartoli C Gentet JC Charbonnel C Gouraud W Voutsinos-Porche B Brouchet A Duffaud F Figarella-Branger D Bouvier C Molecular characterization of the response to chemotherapy in conventional osteosarcomas: predictive value of HSD17B10 and IFITM2 Int J Cancer 2009 1 4 851 860 10.1002/ijc.24457 19449377

[B48] Hawass NED Comparing the sensitivities and specificities of two diagnostic procedures performed on the same group of patients Br J Radiol 1997 1 360 366 9166071 10.1259/bjr.70.832.9166071

